# Puberty classifications in beef heifers are moderately to highly heritable and associated with candidate genes related to cyclicity and timing of puberty

**DOI:** 10.3389/fgene.2024.1405456

**Published:** 2024-06-13

**Authors:** Melanie K. Hess, Anteneh Mersha, Sadie S. Ference, Sarah R. Nafziger, Jessica A. Keane, Anna M. Fuller, Scott G. Kurz, Courtney M. Sutton, Matthew L. Spangler, Jessica L. Petersen, Andrea S. Cupp

**Affiliations:** Department of Animal Science, University of Nebraska–Lincoln, Lincoln, NE, United States

**Keywords:** puberty, heritability, cyclicity, progesterone, FSHR, genomics, beef heifers

## Abstract

**Introduction:** Pubertal attainment is critical to reproductive longevity in heifers. Previously, four heifer pubertal classifications were identified according to attainment of blood plasma progesterone concentrations > 1 ng/ml: 1) Early; 2) Typical; 3) Start-Stop; and 4) Non-Cycling. Early and Typical heifers initiated and maintained cyclicity, Start-Stop started and then stopped cyclicity and Non-Cycling never initiated cyclicity. Start-Stop heifers segregated into Start-Stop-Discontinuous (SSD) or Start-Stop-Start (SSS), with SSD having similar phenotypes to Non-Cycling and SSS to Typical heifers. We hypothesized that these pubertal classifications are heritable, and loci associated with pubertal classifications could be identified by genome wide association studies (GWAS).

**Methods:** Heifers (n = 532; 2017 – 2022) genotyped on the Illumina Bovine SNP50 v2 or GGP Bovine 100K SNP panels were used for variant component estimation and GWAS. Heritability was estimated using a univariate Bayesian animal model.

**Results:** When considering pubertal classifications: Early, Typical, SSS, SSD, and Non-Cycling, pubertal class was moderately heritable (0.38 ± 0.08). However, when heifers who initiated and maintained cyclicity were compared to those that did not cycle (Early+Typical vs. SSD+Non-Cycling) heritability was greater (0.59 ± 0.19). A GWAS did not identify single nucleotide polymorphisms (SNPs) significantly associated with pubertal classifications, indicating puberty is a polygenic trait. A candidate gene approach was used, which fitted SNPs within or nearby a set of 71 candidate genes previously associated with puberty, PCOS, cyclicity, regulation of hormone secretion, signal transduction, and methylation. Eight genes/regions were associated with pubertal classifications, and twenty-two genes/regions were associated with whether puberty was attained during the trial. Additionally, whole genome sequencing (WGS) data on 33 heifers were aligned to the reference genome (ARS-UCD1.2) to identify variants in *FSHR*, a gene critical to pubertal attainment. Fisher’s exact test determined if *FSHR* SNPs segregated by pubertal classification. Two FSHR SNPs that were not on the bovine SNP panel were selected for additional genotyping and analysis, and one was associated with pubertal classifications and whether they cycled during the trial.

**Discussion:** In summary, these pubertal classifications are moderately to highly heritable and polygenic. Consequently, genomic tools to inform selection/management of replacement heifers would be useful if informed by SNPs associated with cyclicity and early pubertal attainment.

## 1 Introduction

Cattle production is the most important agricultural industry in the U.S. comprising the largest share of cash receipts for agricultural commodities (17% of $520 billion dollars for ag commodities) (Economic Research Service, 2023) Profitability is directly related to the ability of producers to develop fertile replacement heifers that have increased reproductive longevity and produce a calf for 3–5 years to replace their heifer development costs (Perry, 2016). Heifers that achieve puberty earlier are more likely to become pregnant, and have a heavier calf at weaning securing their retention in the herd and presumeably increasing reproductive longevity (Gasser et al., 2006; Day and Nogueira, 2013; Perry and Cushman, 2013; Prevatt, 2018) In 2023, heifers comprised 24% of the herd in the U.S. (Economic Research Service, 2023), thus, the ability to identify heifers that will remain in the herd longer would have a dramatic impact on the cow/calf producer and the efficiency of their operation.

A tremendous amount of research has focused on optimizing nutritional management strategies of heifer development (low-cost development, stair-step development, protein supplementation) (Rasby et al., 1998; Gasser et al., 2006; Day and Nogueira, 2013; Cardoso et al., 2014; Amundson et al., 2015; Allen et al., 2017) which has resulted in short-term benefits for the producer. However, many practices that have been implemented to induce early puberty through synchronization with progesterone may have, instead, resulted in the retention of less fertile heifers. If tools were available to interrogate the genetic potential of heifers to reach sexual maturity earlier, then nutritional management could be adjusted to fit their genetic potential. Also, the ability to identify females that genetically are predisposed to delayed puberty would allow for heifers with a decreased likelihood of reproductive success to be culled earlier. The benefit to the producer would be reduced inputs during heifer development and a group of breeding females that are more genetically predisposed to reproductive success.

While genomic regions associated with age at puberty, age at first corpus luteum (CL), first service conception rate, and disorders associated with pubertal attainment, such as polycystic ovary syndrome, (PCOS) have been identified, (Nafziger et al., 2021), no high through-put genomic analysis has been developed to inform producers of what heifers to retain. To start to understand phenotypic and genotypic traits associated with different types of pubertal attainment, and inform potential producer selection, we identified heifer pubertal classifications (Nafziger et al., 2021) in the physiology herd at University of Nebraska. We utilized progesterone concentrations greater than 1 ng/mL with continuous cyclicity in blood plasma collected weekly from weaning to breeding (Oct to May). Initially four pubertal classifications were identified: 1) Early (E), 2) Typical (T), 3) Start-Stop (SS), and 4) Non-Cycling (NC) with E and T heifers initiating and maintaining cyclicity, SS starting and then stopping cyclicity and NC never initiating cyclicity. The Start-Stop heifers were then determined to be two different pubertal groups segregating into Start-Stop-Discontinuous (SSD) or Start-Stop-Start (SSS) with SSD having similar reproductive traits to NC and SSS to T heifers making five pubertal classifications. We hypothesized that these heifer pubertal classifications were heritable, and SNPs could be identified that were associated with puberty and maintenance in females. Understanding genes that contribute to the timing and maintenance of pubertal attainment can inform future research goals and expand our understanding of puberty and fertility-related traits, not only in beef cattle but also in other mammals. Therefore, the goals of this study were to: 1) estimate the heritability of pubertal attainment and maintenance in puberty classifications as determined by circulating progesterone concentration weekly from weaning to breeding; and 2) identify genes and genomic regions associated with pubertal attainment and maintenance.

## 2 Materials and Methods

### 2.1 Ethics, animals and pubertal classifications

The University of Nebraska-Lincoln Institutional Animal Care and Use Committee approved all procedures and facilities used in this manuscript.

A total of 532 beef heifers, born in 2017–2022, were used in this study (Table 1). Heifers were from the physiology herd at the University of Nebraska-Lincoln at the Eastern Nebraska Research, Extension and Education Center (ENREEC). The heifers were a composite comprised of Red Angus and Simmental. Heifers were born in the spring around March and grazed on pasture with dams until weaning in late October. At weaning (6–7 months of age), heifers were separated from dams and male calves and retained as replacement heifers on pasture at ENREEC.

**TABLE 1:** FSHR KASP Genotyping primers.

Variant location	Primer Allele (reference)	Primer Allele (variant)	Primer common
FSHR1-chr11g.31363426 G>A	TGC​TTT​TTT​CGG​CTT​TGT​TTT​AGT​GAT​GC	ATT​GCT​TTT​TTC​GGC​TTT​GTT​TTA​GTG​ATG​T	GGA​TGA​GGG​ATC​ATT​TCC​CAA​CTC​AT
FSHR2-chr11:31404255G G>C	CTT​TCA​GGA​CAA​AGC​CCC​TCT​G	CTT​TCA​GGA​CAA​AGC​CCC​TCT​C	GGC​AGG​GGA​ACA​CAG​CTC​ACA​A

Blood was collected weekly from Oct to May. Progesterone assays were conducted to determine the pattern of progesterone secretion and assigned to pubertal classifications as described previously (Nafziger et al., 2021).

Pubertal classifications were defined and analyzed in four ways: 1) All Levels (**AL**): the five classification levels of E (1), T (2), SSS (3), SSD (4), and NC (5); 2) SSD + NC Combined (**ND**): All Levels, but with SSD + NC combined into one level (4), resulting in four levels; 3) E + T vs NC + SSD (**ETvND**): E + T combined into one level (1), and NC + SSD combined into a second level (2); and 4) E vs T (**ET**): a comparison of only the E (1) and T (2) levels. These 4 analyses explicitly defined different traits and thus posed different questions. Defining the trait complex as in AL and ND estimated genetic components related to the combination of ascertainment of puberty, the timing, and sustained pubertal status. In contrast, reduced subsets such as ETvND and ET enabled the estimation of genetic parameters specific to the timing of pubertal attainment (ET) while the genetic control of puberty attainment during the trial period was estimated using ETvND.

### 2.2 Genotyping and whole genome sequencing

Buffy coats or ear punches were obtained and sent for analysis. DNA was isolated and individuals born between 2017 and 2022 were genotyped (Neogen, Lincoln, NE) on the Illumina BovineSNP50 v2 (54,609 SNPs) or GGP Bovine 100K (95,256 SNPs) SNP panels for variant component estimation and GWAS analyses. There were 32,442 SNPs in common between the two panels. Genotypes that had identical positions were combined and set to missing if the genotypes were not concordant. SNPs from the GGP panel were included in subsequent analyses if they 1) mapped to an autosome on the ARS-UCD1.2 cattle genome assembly, 2) had call rate ≥0.9, and 3) had minor allele frequency ≥0.01. Animals were retained if they had: 1) call rate ≥0.9 and 2) heterozygosity ≤0.5. There were 85,576 markers from 421 animals genotyped on the GGP panel that were phased, and missing genotypes were imputed using BEAGLE v5.1 (Browning, 2018) with default parameters except for Ne = 100. Genotypes were imputed to the retained 85,576 markers for the 484 animals on the Bovine SNP50 array using the phased GGP animals as a reference. After imputation, 532 individuals (265 from BovineSNP50 and 267 from GGP Bovine 100K) with both genotypes and pubertal classifications were retained for further analyses.

Whole genome sequencing (WGS) was conducted on 33 heifers, selected based on completeness of a range of phenotypic records, with identified pubertal classifications (n = 12 NC; n = 15 T, n = 3 E and n = 3 SS) from DNA isolated from potassium-EDTA treated blood, sequenced using 150bp, paired-end reads to a targeted depth of 12X or more. Resulting fastq files were trimmed (TrimGalore!; https://github.com/FelixKrueger/TrimGalore), mapped to the ARS-UCD1.2 genome with BWA-MEM (Li and Durbin, 2009) and duplicates were marked with Samtools (Danecek et al., 2021). GATK was implemented to realign indels, and variants were called using GATK Haplotype Caller (Van der Auwera and O’Conner, 2020). T (Control) and NC (delayed puberty) females were compared to elucidate genes that may contribute to delayed pubertal attainment. From WGS, SNPs within and 10 kb on either side of the genes of interest (Follicle Stimulating Hormone Receptor-*FSHR*; Follicle Stimulating Hormone beta- *FSHb*; Anti-Mullerian Hormone- *AMH*; Anti-Mullerian Hormone Receptor 2- *AMHR2*) were extracted. These genes were previously determined to affect follicular development/arrest, response to Follicle Stimulating Hormone (FSH) stimulation, puberty and PCOS in humans and livestock (Wunsch, Sonntag, and Simoni, 2007; Cory et al., 2013; Simoni and Casarini, 2014; Jones and Goodarzi, 2016; Wang et al., 2017; Yang et al., 2018; Tahir et al., 2021; Ramesha et al., 2022).

### 2.3 Heritability estimates

Univariate animal models were implemented in JWAS v1.6.1 (Cheng, Fernando, and Garrick, 2018) in a Bayesian framework using the following model:

y=Xb+Zu+e, (1)

where **y** is a vector of the ordinal categorical pubertal classifications following the numbers specified in 2.1 (by specifying it as a categorical_trait in build_model), **b** is a vector of fixed effects, **u** is a vector of random additive genetic effects, **e** is a vector of random residuals and **X** and **Z** are design matrices relating observations to the fixed and random additive genetic effects, respectively. Fixed effects included the intercept, year of birth, and a covariate of birth date deviation (individual birth date minus the average birth date for that birth year). The random additive genetic effect was assumed ∼ N(0, $\left.{G\sigma }_{u}^{2}\right)$ , where G is a genomic relationship. The **G** matrix was constructed as:

$$G=\frac{MM^{\prime }}{2\sum p_{j}\left(1-p_{j}\right)},$$

where **M** is a genotype incidence matrix that has been centered based on allele frequencies (VanRaden, 2008) and *p* is the allele frequency of the second allele at the *j*th SNP across all loci. Given the categorical nature of the phenotypes, the residual variance was set to 1.0. Priors were the default priors for JWAS, which includes flat priors for fixed effects and a default prior for heritability of 0.5. The model was run for a chain length of 100,000 with the first 25,000 samples discarded as burn-in. Estimates of genetic variance and heritability were the average of the 750 Markov Chain Monte Carlo (MCMC) samples output from the model (1 in every 1,000 samples). Convergence was evaluated based on trace plots of variances and heritability estimates, and also by testing a range of priors for heritability to evaluate the sensitivity of the results to different priors.

### 2.4 Genome wide association

A Genome Wide Association Study (GWAS) was conducted using JWAS v1.6.1 (Cheng et al., 2018) using a BayesB model (Meuwissen, Hayes, and Goddard, 2001) with the same fixed effects as described in model 1 above. For the GWAS, the traits considered were AL, ET, and ETvND. The BayesB model employed allows for locus-specific variances, thus enabling local shrinkage of SNP effects. The proportion of the 85,576 SNP assumed to have a null effect, π, was set to 0.99 *a priori*. Priors for the genetic variance were set to the posterior estimates from the GBLUP models described above, and fixed effects had flat priors. The model was run for a chain length of 100,000 with the first 25,000 samples discarded as burn-in. Results from the GWAS are presented in a Manhattan plot, with the proportion of variance explained by each 1 Mb window on the y-axis, and points sized based on the window posterior probability of association (WPPA). We considered a window to be associated with a trait if it explained more than 1% of the genetic variation of that trait.

### 2.5 Candidate gene and *FSHR* SNP analysis

Previous studies have identified genes associated with pubertal attainment in humans, cattle, and other species (Hayes et al., 2015; Busch et al., 2016; Fortes et al., 2016; Stegemiller et al., 2021; Tahir et al., 2021). We collated a set of 71 of these genes that were on autosomes in cattle according to the ARS UCD 1.2 *Bos taurus* genome assembly (https://genome.ucsc.edu). Our set of candidate SNPs were within the 71 genes, or, if there were no SNPs within the gene, SNPs within 10,000 bases of the gene were considered. In total, 389 SNPs within or near the set of 71 genes were studied (Supplementary File S1).

Effects of the 389 SNP were estimated by fitting model 1 with the addition of fixed covariates for each SNP, one at a time, coded as 0,1,2. The posterior mean and standard deviation of SNP effects were determined by 750 of the 75,000 MCMC samples (those that were output) and the pseudo-p-value was calculated as $2\times \min\left(s,1-s\right)$ where *s* was the proportion of MCMC samples where the sampled SNP effect was less than zero (Shi and Yin, 2021).

Due to FSHR’s importance in fertility, (Gloaguen, 2011; Cory et al., 2013; Busch et al., 2016; Nijeeb, Alkazaz, and Yaseen, 2020), SNPs were identified using Fisher exact test that had significant association with NCvT, and were absent on the bovine SNP panel. Two novel loci within *FSHR,* identified in our population through WGS, were chosen for further genotyping in 308 additional individuals: FSHR1, chr11:31363426G>A, and FSHR2, chr11:31404255G>C (rs21971504). Kompetitive Allele Specific PCR (KASP) genotyping was conducted using primers and probes designed with the KASP on Demand utility (LGC Genomics, Teddington, Middlesex, United Kingdom; Table 1). All KASP reactions were performed in duplicate on a CFX384 Touch Real-Time PCR machine (Bio-Rad, Hercules, California) following the manufacturer’s protocol. Non-template (negative) controls, homozygous reference controls, heterozygous controls, and homozygous alternative controls were run on each plate from the initial 33 cattle with WGS data. Results were visualized on the CGX Maestro Software (Bio-Rad Laboratories, Hercules, CA, United States). Both loci were analyzed for their association with the pubertal classification, using the same model and method as described, in a total of 308 individuals.

## 3 Results

### 3.1 Heritability estimates for pubertal classifications and cyclicity during trial

Table 2 depicts the number and percentage of individuals assigned to each pubertal classification class, separated by birth year. Overall, the SSS and SSD classes were the rarest, followed by NC, then E, with T being the most common classification. The percentage of animals allocated to each classification varied across the years for all classes (Fishers Exact test on counts: *p*-value = 0.0005).

**TABLE 2:** Incidence (percent) of individuals assigned to each pubertal classification by birth year.

Year	Early	Typical	SSS^a^	SSD^b^	Non-cycling	Total^c^
2017	29 (38)	35 (46)	0 (0)	2 (3)	10 (13)	76 (14)
2018	30 (37)	27 (33)	16 (20)	5 (6)	3 (4)	81 (15)
2019	17 (16)	59 (55)	6 (6)	4 (4)	22 (20)	108 (20)
2020	16 (17)	40 (42)	8 (8)	6 (6)	25 (26)	95 (18)
2021	34 (43)	22 (28)	12 (15)	6 (8)	6 (8)	80 (15)
2022	23 (25)	43 (47)	13 (14)	8 (9)	5 (5)	92 (17)
Total	149 (28)	226 (42)	55 (10)	31 (6)	71 (13)	532 (100)

a SSS: Start-Stop-Start.

b SSD: Start-Stop-Discontinued.

c Percentages are the percent of the total dataset that is accounted for by that birth year.

**FIGURE 1:**
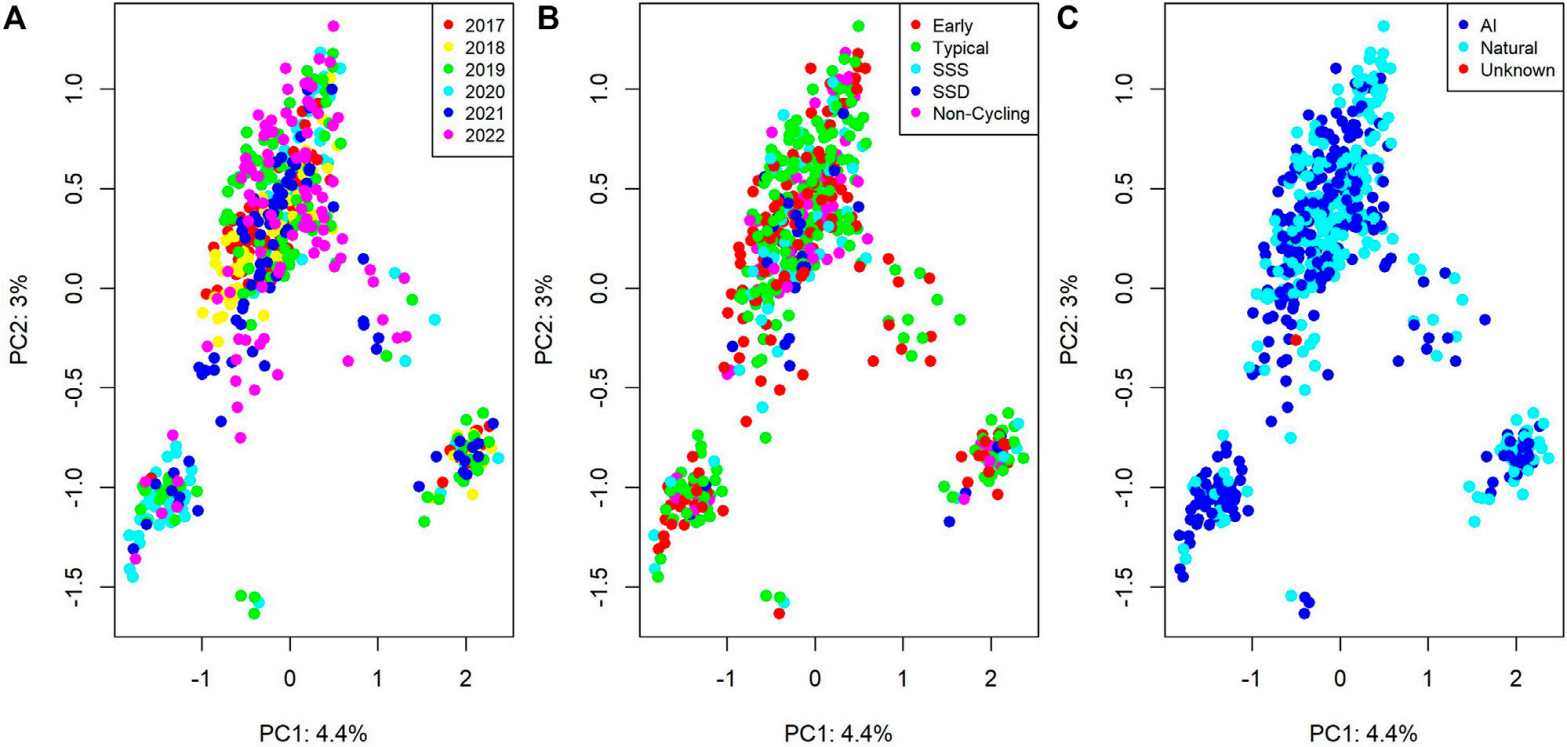
Genomic relationships between individuals from a principal component analysis. Plots colored by **(A)** birth date, **(B)** pubertal classification (SSS = Start-Stop-Start; SSD = Start-Stop- Discontinued), and **(C)** sire type (AI = Artificial Insemination; Natural = Natural Mating).

Figure 1 visualizes the first two principal components of the genomic relationship matrix, colored by birth year, pubertal classification, and whether the individual was conceived via artificial insemination (AI) or natural mating.

Table 3 identifies that heritability estimates for these traits were moderate to high. Heritability estimates of AL and ND were similar with AL being slightly greater, therefore, AL was used remaining analyses. The heritability of ETvND was the greatest of all the traits. The heritability estimate of E vs T classifications was less than that for the other traits and not significantly different from zero.

**TABLE 3:** Heritability estimates for different pubertal classification comparisons.

Comparisons	Number of levels	Genetic variance	Total variance	Heritability (95% credible Set)
All Levels	5	0.64 ± 0.21	1.64 ± 0.21	0.38 ± 0.08 (0.23–0.53)
SSD and NC Combined	4	0.57 ± 0.21	1.57 ± 0.21	0.35 ± 0.08 (0.21–0.51)
Early + Typical vs Non-Cycling + SSD	2	2.54 ± 3.50	3.54 ± 3.50	0.59 ± 0.19 (0.22–0.92)
Early vs Typical	2	0.29 ± 0.36	1.29 ± 0.36	0.19 ± 0.14 (0.03–0.55)

### 3.2 GWAS

**FIGURE 2:**
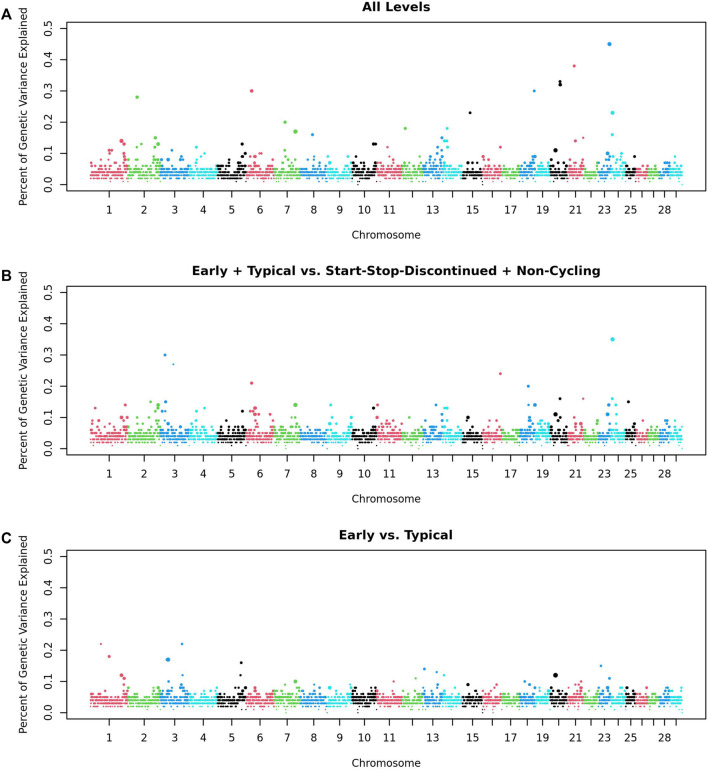
Genome Wide Association Analysis of Pubertal Classifications. GWAS of **(A)** All five trait levels; **(B)** Early and Typical vs Start-Stop-Discontinued (SSD) and Non-Cycling (2 levels); and **(C)** Early vs Typical (2 levels). Each point represents a 1 Mb window. Points are colored by Chromosome and sized based on the Window Posterior Probability of Association (WPPA).

Within the GWAS of 532 heifers, no regions were significantly associated with any of the traits- AL, ETvND or EvT: the greatest percentage of genetic variance explained by a 1 Mb window was less than half a percent, and the greatest Window Posterior Probability of Association (WPPA) was 0.30 (Figure 2). We considered a window to be associated with a trait if it explained more than 1% of the genetic variation of that trait as stated in the Materials and Methods.

The top 10 windows from the GWAS for each trait were evaluated to determine their distance from the closest candidate gene(s). There were four windows that were within 5 Mb of one of the candidate genes: naturietic peptide receptor 3 (*NPR3;* AL), *NDUFA2* (AL), *BORCS5* (ET and ETvND), and Mitogen-Activated Protein Kinase Kinase 1 (*MAP3K1*; ET and ETvND).

### 3.3 Candidate genes that were associated with pubertal classification or cyclicity

**FIGURE 3:**
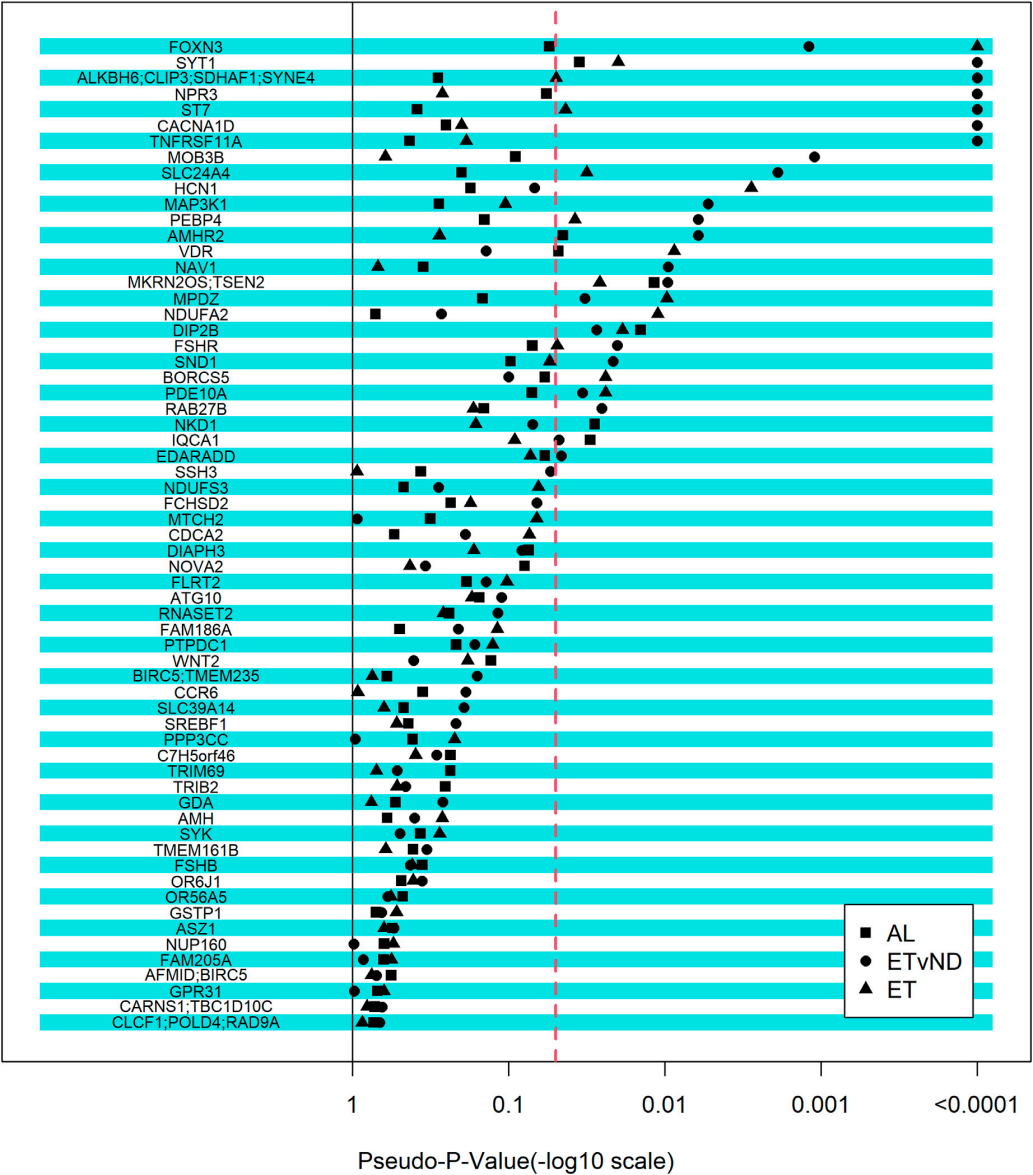
Strength of association between candidate genes and pubertal classification traits. The -log10(pseudo-p-value) equal to 4 relates to a pseudo-p-value of zero. AL = All Levels (Early, Typical, Start-Stop-Start, Start-Stop-Discontinued, Non-Cycling); ETvND = Early and Typical vs Start-Stop-Discontinued and Non-Cycling; and ET = Early vs Typical. Dotted line represents a pseudo-p-value of 0.05.

Although we did not identify any regions that were associated with the pubertal classification traits in the GWAS, a candidate gene approach was used, which relied on external information to study genes associated with puberty, associated puberty traits or PCOS in humans, and cattle. The selected set of genes were fitted as fixed effects, with one SNP fitted at a time. Twenty-seven genomic regions were associated with AL, ET and/or ETvND (Pseudo-p-value <0.0001; Figure 3 and Table 4). Four gene regions were associated with all three groups.

**TABLE 4:** Candidate genes associated with pubertal classifications.

Chr	Mb	AL	ET	ETv ND	Candidate Gene(s)	Function	Citation
3	115	X		X	*IQCA1*	Fasting glucose levels (plasma)	Wang et al. (2023)
4	51		X	X	*ST7*	Hormone exocytosis	Battle et al. (2003)
4	92			X	*SND1*	Cholesterol synthesis	Armengol et al., 2017; Navarro-Imaz et al., 2020
5	8	X	X	X	*SYT1*	Neuronal growth and development	Baker et al., 2018; Riggs et al., 2022
5	26	X		X	*AMHR2*	Receptor for AMH. Puberty and ovulation	Sacchi et al., 2016; Ramesha et al., 2022
5	29	X	X	X	*DIP2B*	Neuronal growth and development	Xing et al. (2020)
5	32	X	X		*VDR*	Inflammation and reduction of tissue repair	Tourkochristou et al. (2023)
5	97		X		*BORCS5*	Neuronal activity and transport	De Pace et al. (2020)
7	51		X		*NDUFA2*	Regulates luteinizing hormone production	Li et al. (2021)
8	16			X	*MOB3B*	Hippo pathway inhibitor	Joung et al. (2017)
8	31		X	X	*MPDZ*	Angiogenesis	Tetzlaff et al. (2018)
8	70		X	X	*PEBP4*	Cattle growth and fertility	Kour et al. (2023)
9	100		X	X	*PDE10A*	Oocyte maturation, inhibits cAMP	Tahir et al. (2021)
10	100		X	X	*FOXN3*	WNT signal transduction pathway	Zhao et al. (2020)
11	31	X		X	*FSHR*	Fertility and delayed puberty	Pyun et al. (2014)
16	80			X	*NAV1*	Neurite outgrowth and development	Maes et al. (2002)
18	18	X			*NKD1*	WNT signaling pathway	Katoh, (2001)
18	46	X	X	X	*ALKBH6*	Nucleic acid damage repair	Ma et al. (2022)
					*CLIP3*	Glycolysis	Kang et al. (2021)
					*SDHAF1*	Mitochondria and TCA cycle	Fullerton et al. (2020)
					*SYNE4*	Deafness	Worman and Segil, (2013)
20	22			X	*MAP3K1*	Development of the female reproductive tract	Alaniz et al., 2015; Kimura et al., 2023
20	29		X		*HCN1*	Initiates GnRH surge causing ovulation, voltage gated- ion channel	Arroyo et al., 2006; Zhang et al., 2013
20	40			X	*NPR3*	Found on gonadotroph cells	Mirczuk et al. (2019)
21	57		X	X	*SLC24A4*	Calcium Transport	Parry et al., 2013; Thibodeau et al., 2021
22	47			X	*CACNA1D*	Cardiac pacemaking and hormone secretion	Pinggera and Striessnig, (2016)
22	56	X	X	X	*MKRN2OS*	Increased inflammation	Wang et al. (2022)
					*TSEN2*	tRNA processing and splicing	Hayne et al. (2023)
24	54			X	*RAB27B*	Pituitary hormone exocytosis	Zhao et al. (2002)
24	60			X	*TNFRSF11A*	Menarche and menopause	Lu et al. (2010)
28	9			X	*EDARADD*	MAPK signaling pathways	Fang et al. (2022)

AL = All Levels (Early, Typical, Start-Stop-Start, Start-Stop-Discontinued, Non-Cycling); ETvND = Early and Typical vs Start-Stop-Discontinued and Non-Cycling; and ET = Early vs Typical.

### 3.4 *FSHR* associations with pubertal classification or cyclicity

Two novel SNPs in the *FSHR* gene that we named FSHR1 and FSHR2 were identified via WGS that segregated to NC compared to T groups. These SNPs were also not present on bovine SNP arrays that were used to genotype the herd. Linkage disequilibrium between the two SNPs genotyped in *FSHR* was determined to be moderate (r = 0.476). The FSHR1 SNP was not associated with any of the pubertal classification traits (*p* > 0.12); however, FSHR2 was associated with AL and ETvND traits (*p* < 0.01), but not ET (*p* > 0.6; Table 5 and Figure 3).

**TABLE 5:** *FSHR* KASP genotyping analysis.

SNP name	SNP position (chr 11)	Trait^1^	MAF	Effect estimate	Pseudo-P-Value
FSHR1	31,363,426	AL	0.39	0.23 ± 0.15	0.1154
		ETvND	0.38	3.02 ± 2.41	0.2208
		ET	0.39	−2.91 ± 4.45	0.4992
FSHR2	31,404,255	AL	0.36	0.32 ± 0.14	0.0094
		ETvND	0.32	6.18 ± 2.80	0.0008
		ET	0.36	1.07 ± 2.11	0.6074

1) AL = all levels; ETvND = Early and Typical vs Start-Stop-Discontinued and Non-Cycling; ET = Early vs Typical.

## 4 Discussion

### 4.1 Heritability of puberty classifications

The goals of this study were to estimate the heritability of puberty classifications and determine whether we could identify SNPs associated with the trait. Accurate genomic predictions, available prior to heifer development, could replace weekly blood samples from weaning to breeding to measure progesterone, thus, providing a less labor-intensive method of selecting heifers that were genetically predisposed to earlier age at puberty.

Puberty classifications in the current experiment were moderately to highly heritable, which supports further study into the genes and pathways responsible for pubertal attainment. Heritability was estimated to be moderate to high in the UNL Physiology herd in all five pubertal groups (AL; moderately heritable), four groups (with SSD and NC combined = ND; moderately heritable), or groups that attained puberty and maintained cycling, compared to those that never reached puberty or failed to maintain cycling (ETvsND; highly heritable). Heritability estimates for AL and ND were numerically similar. The lowest heritability estimate was for ET which would have distinguished between early and T puberty (E vs T) for those individuals that attained puberty and maintained cycling. Despite relatively large credible sets for the heritability estimates, particularly for ET and ETvND, none of the credible sets included zero, indicating that we can be confident that the pubertal classifications are heritable. The confidence in our estimates based on both standard errors and credible sets is consistent with what might be expected for running a complex (ordinal) model on a dataset of this size. These results suggest that attaining and maintaining, puberty ETvND and all five puberty classifications (AL) would respond favorably to selection enabling the development of genomic selection tools to aid producers in improving fertility and decreasing heifer development costs.

Estimates of heritability for traits related to age at puberty exist in the literature including age at first CL (Engle et al., 2019), age at first conception (Pereira et al., 2006), or first calving (Dubon et al., 2021). Many of these traits can impact age at mating, and age at birth (calving or farrowing) (Li et al., 2018; Dubon et al., 2021). Several studies have reported that age at puberty is lowly to moderately heritable in cattle (0.1–0.38, (Fortes et al., 2016; Lefebvre et al., 2021; Stephen et al., 2023a), and pigs (0.25–0.42; (Tart et al., 2013; Li et al., 2018), in agreement with the estimates from the current study when all five classifications were used. In addition to additive genetic variation, environmental and management effects such as yearly variation in weather, heat stress and management decisions including differences in AI and natural mating sires and nutritional supplementation by the producer can play a pivotal role in pubertal differences among animals (Patterson and Smith, 2013). In our population of heifers, the proportion of females assigned to each of the puberty classifications differed by year (Nafziger et al., 2021). Furthermore, in the current study there was an increased number of females assigned to the NC puberty classification in 2019 and 2020. Both of those years had dramatic weather changes with excessive rainfall in 2019 and drought in 2020. Thus, harsh weather conditions can impact genetic predisposition and cause delayed puberty in heifers.

While age at puberty measured in other studies are like those of our comparison of E and T heifers, there have been studies where small numbers of progesterone samples have been collected and/or only initial rise in progesterone has been used to determine age at puberty (Lefebvre et al., 2021; Stephen et al., 2023a). Consequently, the heritability estimates from these sources may have been for a different definition of the trait given in the current study we observed two different Start-Stop groups within our puberty classifications. The SSS heifers reproductive performance appears to be similar to that of T heifers (Nafziger et al., 2021) so inability to identify this group may not be critical. However, SSD heifers present a problem as they do not maintain cyclicity during the experiment and have reduced reproductive performance, similar to that of the NC females (Nafziger et al., 2021). The SSD and NC groups also have a reduced percentage of females with a reproductive tract score of 5, which suggests they are sexually immature and have reduced numbers of calves within the first 21 days of the calving season. These reproductive performance parameters indicate that heifers in the NC and SSD pubertal classifications would be predisposed to a reduced reproductive lifespan. Growth was also reduced in these two pubertal classifications with lower adjusted yearling weight when compared to E and T heifers (Nafziger et al., 2021). Within the UNL physiology herd, NC (delayed puberty) heifers comprise approximately 16% of the herd with 6% in the “false start” SSD group (Nafziger et al., 2021). Similar numbers were observed in the current study with 13% NC and 6% SSD from heifers categorized between 2017 and 2022. To be able to select against the 19%–22% of the heifers in the herd with delayed or “a false start” puberty would decrease the average age at puberty within the herd and increase the sustainability of beef cattle operations.

### 4.2 Relationship between puberty classifications and high A4 cows

The development of puberty classifications in the UNL Physiology herd also allowed us to determine the differences in how heifer puberty was attained in these females and to potentially predict which heifers would become our naturally occurring High Androstenedione (High A4) population (Summers et al., 2014). We have identified a naturally occurring androgen excess (High A4) population within our research herd and have also identified similar populations of bovine and ovine females in the US and Middle East with an incidence of 18%–26% which may contribute to impaired follicular development and ovulatory failure in livestock world-wide. (Summers et al., 2014; Abedal-Majed et al., 2022a; Abedal-Majed et al., 2022b). In addition to androgen excess, these High A4 females also have increased inflammatory markers, fibrosis, and follicular arrest, all of which may contribute to anovulation and infertility (Abedal-Majed et al., 2022a). High A4 cows and sheep have similar characteristics to women diagnosed with polycystic ovary syndrome (PCOS) (Summers et al., 2014; Abedal-Majed and Cupp, 2019; Abedal-Majed et al., 2019; McFee et al., 2021; Abedal-Majed et al., 2022a; Abedal-Majed et al., 2022b). The disorder, PCOS, is strongly familial and the prevalence rate is 21% across many human populations and is highly heritable. Often 60% of daughters with mothers diagnosed with PCOS also have this disorder (Stener-Victorin et al., 2020). Women with PCOS are often diagnosed at puberty due to irregular, precocious or delayed puberty (Rosenfield, 1991; Rosenfield et al., 2000; Nicandri and Hoeger, 2012; Rosenfield et al., 2015b). Heifers from the NC group also have been observed to have some of the characteristics of High A4 cows and we are investigating the potential that they are a portion of this population. Thus, current pubertal classifications provide unique information that may allow us to predict not only who should be retained in the herd but allow us to evaluate alterations in factors causing excess steroidogenesis resulting in follicle arrest, infertility and potentially precocious or delayed puberty.

### 4.3 GWAS and candidate genes

Despite our pubertal classifications being moderately to highly heritable, no genomic regions were significantly associated with pubertal classifications in our GWAS. This indicates that the pubertal classifications are polygenic traits. Other studies that have tried to define regions of interests through GWAS analysis specific to puberty onset used a greater number of females and utilized other traits associated with age at puberty or female fertility such as: anogenital distance (Stephen et al., 2023b), antral follicle counts (Stegemiller et al., 2021), age at puberty in dairy (Lefebvre et al., 2021) and beef (Vargas et al., 1998), age at first CL in *Bos indicus* (Fortes et al., 2012), and age at menarche, (Perry et al., 2014). While other studies have combined RNAseq data and other “omics” from different reproductive tissues obtained at or around puberty (Cánovas et al., 2014; Tahir et al., 2021; 2022). The novelty of the current study is that this is the first-time pubertal classifications have been developed using extensive progesterone concentrations from weaning to breeding to address phenotype to genotype associations in beef heifers. Furthermore, our pubertal classifications not only evaluate first ovulation (first rise in progesterone >1 ng/mL) but also indicate whether puberty is maintained (Nafziger et al., 2021), which may not be considered in other studies (e.g., Lefebvre et al., 2021; Stegemiller et al., 2021).

To further investigate our genomic data, a candidate gene approach using 389 SNPs in or near 71 candidate genes that were involved in previous studies of puberty, reproductive parameters surrounding puberty, PCOS, signal transduction, regulation of calcium, and hormonal secretion was conducted (Cory et al., 2013; Hagen et al., 2013; Cánovas et al., 2014; Hayes et al., 2015; Fortes et al., 2016; Ali et al., 2017; Dapas et al., 2020; Nijeeb et al., 2020; Tahir et al., 2021). Of the 71 candidate genes, SNPs in/near 13 genes were associated with AL, 18 with ET and 25 with ETvND. Not surprisingly, genes regulating FSH’s functions were associated with different pubertal group comparisons, along with genes regulating calcium mobilization, metabolism, hormone secretion and signal transduction.

Anti-Mullerian Hormone receptor 2 (*AMHR2*), a specific receptor for AMH, acts to suppress FSH through its receptor FSHR. Increased AMH concentrations in circulation is proposed to be a measure of ovarian reserve and increased fertility (Van Houten et al., 2010; Sacchi et al., 2016; Ramesha et al., 2022) and AMH secretion has been shown to be reduced as heifers achieve puberty which may be necessary for follicle progression to ovulation. Another gene that may indirectly affect FSH’s action through AMH is the vitamin D receptor (*VDR*). Mutations of the *VDR* can affect AMH function and steroidogenesis (Merhi et al., 2014). Vitamin D acting through VDR can inhibit AMH secretion, and reduce production of excess steroids as seen in women diagnosed with PCOS (Bakhshalizadeh et al., 2017). Thus, altered function of the VDR can increase AMH secretion which can cause follicular arrest by inhibiting the actions of FSH which may contribute to delayed puberty. Conversely, mutations in the *VDR* gene can cause inflammation through fibrosis of the liver and alterations in immune macrophage function (Tourkochristou et al., 2023).

The FSHR, a G-protein coupled receptor, is expressed in granulosa cells which surround the developing oocyte within the ovary. The *FSHR* gene and SNPs associated with it have been found to be predictive of fertility, delayed puberty and infertility in humans, and cattle (Pyun et al., 2014; Tahir et al., 2021). No FSHR SNPs on the bovine SNP arrays used in the current study were found to be associated with any pubertal classification parameters. Thus, we utilized our WGS to investigate novel SNPs present in our herd. There were two SNPs found with WGS that were not within the bovine SNP arrays. One of the SNPs associated with all puberty classifications (AL) and the ability of heifers to become pubertal and maintain cyclicity (ETvND). For the purposes of this study, we named this SNP FSHR2. The FSHR2 SNP had a more significant association with AL and ETvND than any of the FSHR SNPs on the bovine SNP arrays used in this study, therefore, inclusion of this SNP (FSHR2) in future bovine SNP arrays may improve prediction of fertility traits.

Regulation of FSH secretion occurs via the hypothalamus and actions of neurons that secrete GnRH. Most of this regulation involves neuronal excitation and calcium mobilization and activation of second messenger signal transduction pathways. The hyperpolarization activated cyclic nucleotide voltage gated potassium channel 1 (*HCN1*) gene encodes a protein that is in the superfamily of voltage-gated ion channels and appears to have a major role in controlling neuronal excitability and is positively regulated by cyclic AMP which means that G-protein-coupled receptors regulate these ion channels and the amount of calcium that comes into the cell. GnRH binds to G-protein coupled GnRH receptors. These HCN channels are on both GnRH neurons and kisspeptin neurons. The hormone, 17beta-estradiol, can stimulate burst firing of these HCN1 channels within Kiss neurons to initiate their secretion which acts on GnRH neurons to cause surge levels of GnRH and ovulation (Arroyo et al., 2006; Zhang et al., 2013). The calcium voltage-gated channel subunit alpha1 D (*CACNA1D*) gene also encodes a G protein activated calcium channel and SNPs within this gene have shown to cause aberrant release of hormones (Pinggera and Striessnig, 2016). Variants in the *CACNA1D* gene have been shown to associate with enhanced reproduction within two breeds of zebu cattle (Sukhija et al., 2024) that are climate resilient.

The *NPR3* gene is found on gonadotroph cells within the anterior pituitary and gonadotropin releasing hormone (GnRH) pulsatile secretion increases secretion of the peptide (Mirczuk et al., 2019) which may influence release of luteinizing hormone (LH) and FSH. Alternatively, the phosphodiesterase 10A (*PDE10A*) gene is a phosphodiesterase that inactivates cAMP inhibiting G-protein-coupled signal transduction from the hypothalamic (GnRH) and anterior pituitary hormones (FSH and LH) and attenuates the signal allowing for receptor downregulation (Yang et al., 2018). The *PDE10A* gene has previously been identified to contain a SNP which was associated with pubertal attainment in heifers (Tahir et al., 2021). The member RAS oncogene family (*RAB27B*) (Zhao et al., 2002) and Suppressor of tumorigenicity protein 7 (*ST7*) genes (Battle, Maher, and McCormick, 2003), are most abundantly expressed in the pituitary and involved in regulation of exocytosis of pituitary hormones (Zhao et al., 2002), Thus, alterations in their functions would also result in too little hormone being released to affect the ovary. Mutations in all of these genes regulating calcium mobilization, exocytosis of pituitary hormones could all contribute to either heifers attaining early or delayed puberty and should be studied further to determine their potential in these puberty classifications.

There were several genes that affect beta catenin and the WNT signal transduction pathway. Overexpression of Forkhead box protein N3 Forkhead box protein N3 (*FOXN3*) has been shown to inhibit the WNT signal transduction pathway (Zhao et al., 2020) which can affect beta catenin and regulation of cell growth in granulosa cells which could cause follicular arrest. The gene NKD Inhibitor of WNT Signaling Pathway (*NKD1*) is a dishevelled-binding protein, functioning as a negative regulator of WNT - beta-catenin - TCF (T-cell factor/lymphoid enhancer factor) signaling pathway (Katoh, 2001). The WNT-beta catenin pathway is involved in reproductive functions including development of gonadal sexual morphogenesis and regulation of follicle maturation and steroidogenesis within the ovary (Hernandez Gifford, 2015) which could affect the ability of puberty to occur early vs late. Another signal transduction pathway that has been shown to affect development of the reproductive tract is MAP3K1. Mutations in *MAP3K1* have been associated with imperforate vagina, labor failure, infertility, and in Swyer syndrome, where genetic males appear to physically be females, in humans and mice (Alaniz et al., 2015; Yu et al., 2022; Kimura et al., 2023). These developmental problems which range from severe to slight could be physiological explanations for how *MAP3K1* could be associated with cattle not attaining or maintaining puberty.

There were four genomic regions that were associated with the three pubertal groups tested in the current study (AL, ET and ETvND): Chr5_8 Mb synaptotagmin-1(*SYT1*), Chr5_29 Mb DIP2B disco interacting protein 2 homolog B (*DIP2B*), Chr18_46 Mb {alpha-ketoglutarate-dependent dioxygenase-6 (*ALKBH6*)*, C*AP-Gly Domain Containing Linker Protein 3 (*CLIP3*), succinate dehydrogenase complex assembly factor 1 (*SDHAF1*), and spectrin repeat containing nuclear envelope family member 4 (*SYNE4*)}, and Chr22_56 Mb { makorin ring finger protein 2 (*MKRN2OS*), TRNA Splicing Endonuclease Subunit 2 (*TSEN2*)}. These genes are involved in many different functions that can be involved in pubertal attainment including regulation of anterior pituitary cells, neuronal development, hormone secretion, methylation or demethylation of genes or altering inflammation (see Table 4). These four regions should be evaluated further to determine if they are critical for certain aspects of pubertal attainment in heifers.

### 4.4 Conclusions and implications

Through weekly progesterone measurements during the postweaning period we defined multiple granular definitions of pubertal status in developing *B. taurus* females. All these traits were estimated to be moderately to highly heritable, suggesting that they would respond favorably to selection ultimately reducing the frequency of delayed or precocious puberty. The FSHR2 SNP identified in this study was associated with the pubertal classifications and whether heifers attained and maintained puberty during the trial. The addition of this FSHR2 SNP to future SNP arrays may improve genetic predictions for pubertal status and allow for identification of heifers that are predisposed to attain and maintain puberty. Finally, many different candidate genes containing SNPs were associated with the pubertal classifications or pubertal groups we tested within the current study. Further investigation into the physiological processes that are represented by these genetic variants around or within these genes may allow for development of better tools to retain females within the herd that have greater reproduction longevity due to a predisposition to earlier puberty.

## Data Availability

The original contributions presented in the study are publicly available. The Whole Genome Sequence data are available in the NCBI SRA under Accession PRJNA1042814 [https://www.ncbi.nlm.nih.gov/bioproject/?term=PRJNA1042814]. Genotypes and phenotypes are available in the Open Science Framework at: https://osf.io/cygx7/.
